# Coordination chemistry of nitrile-functionalized mixed thia-aza macrocycles [9]aneN_2_S and [9]aneNS_2_ towards silver(I)

**DOI:** 10.1107/S205322962200105X

**Published:** 2022-02-14

**Authors:** Alexander J. Blake, Vito Lippolis, Martin Schröder

**Affiliations:** a School of Chemistry, University of Nottingham, University Park, Nottingham, NG7 2RD, UK; b Dipartimento di Scienze Chimiche e Geologiche, Università degli Studi di Cagliari, S.S. 554 Bivio per Sestu, Monserrato (CA), 09042, Italy; c Department of Chemistry, The University of Manchester, Manchester, M13 9PL, UK

**Keywords:** macrocylic ligands, silver(I), thio­cyanate ligand, nitrile derivatives, crystal structure

## Abstract

The coordination chemistry towards silver(I) of the small-ring macrocycles [9]aneN_2_S (1-thia-4,7-di­aza­cyclo­nona­ne) and [9]aneNS_2_ (1,4-di­thia-7-aza­cyclo­nona­ne) incorporating nitrile-functionalized pendant arms is considered both in the presence and the absence of exogenous bridging ligands. The aim is to understand the influence of the number and length of the pendant arms on the nuclearity and dimensionality of the resulting com­plexes. The X-ray crystal structures of the com­plexes bis­[4,7-bis­(cyano­meth­yl)-1-thia-4,7-di­aza­cyclo­non­ane-κ^3^ *N*,*N*′,*S*]silver(I) tetra­fluorido­borate, [Ag(C_10_H_16_N_4_S)_2_]BF_4_ or [Ag(**L^1^**)_2_]BF_4_, [4,7-bis­(2-cyano­eth­yl)-1-thia-4,7-di­aza­cyclo­nonane-κ^3^ *N*,*N*′,*S*](thio­cyanato-κ*S*)silver(I), [Ag(C_12_H_20_N_4_S)(NCS)] or [Ag(**L^2^**)(SCN)], and μ-thio­cyanato-κ^2^ *S*:*S*-bis­{[7-(2-cyano­eth­yl)-1,4-di­thia-7-aza­cyclo­nonane-κ^3^ *N*,*S*,*S*′]silver(I)} tetra­fluor­ido­borate, [Ag_2_(C_9_H_16_N_2_S_2_)_2_(SCN)]BF_4_ or [Ag_2_(**L^3^**)_2_(μ-SCN)]BF_4_, are discus­sed in relation to analogous com­pounds in the literature.

## Abstract 2

The aim of this study is to determine how far the length and number of the pendant arms in nitrile-functionalized derivatives of small mixed-donor macrocycles influence the structure of silver(I) com­plexes, both in the presence and absence of exogenous ligands.

## Introduction

In previously published articles (Tei *et al.*, 1998 ▸, 2002 ▸), we have considered the nitrile-functionalized pendant-arm derivatives of mixed-donor macrocycles as multidentate ligands for the syn­thesis of multidimensional polymeric com­plexes with silver(I). We argued that nitrile-containing pendant arms would promote exocyclic rather than endocyclic com­plex­ation, thereby preventing the formation of mononuclear com­plexes in favour of coordination polymers. The results confirmed this hypothesis, with the nitrile groups playing an active role in linking different silver(I) centres in the obtained polynuclear com­plexes whose dimensionality is strictly de­pen­dent upon the number of nitrile-functionalized pendant arms present in the ligand, upon their length, and upon the donor set and ring size of the macrocyclic framework. However, in the presence CN^−^, the coordination site left free on the metal centre by the macrocyclic moiety of the nitrile-functionalized ligands in Scheme 1 [**L^1^** = 4,7-bis­(cyano­meth­yl)-1-thia-4,7-di­aza­cyclo­nonane, **L^2^** = 4,7-bis­(2-cyano­eth­yl)-1-thia-4,7-di­aza­cyclo­nonane and **L^3^** = 7-(2-cyano­eth­yl)-1,4-di­thia-7-aza­cyclo­nonane] was occupied by the exogenous anionic ligand instead of nitrile groups, thus preventing the formation of inorganic polymers involving the pendant nitriles and favouring the isolation of unusual com­pounds (Lippolis *et al.*, 1999 ▸; Blake *et al.*, 1998 ▸).

In particular, while the discrete binuclear com­plex [Ag_2_(**L^1^**)_2_(μ-CN)]BF_4_·MeCN, featuring a side-on two-electron (σ) μ_2_-κ*C*:κ*C* bridging cyanide, was isolated from the reaction of **L^1^**, AgBF_4_ and ^*n*^Bu_4_NCN in a 1:1:0.5 molar ratio, the com­plexes [Ag_2_(**L^2^**)_2_(μ-CN)]BF_4_ and [Ag_2_(**L^3^**)_2_(μ-CN)]BF_4_, exhibiting a CN^−^ ligand bridging two metal centres in a linear four-electron (σ + π) μ_2_-κ*C*:κ*N* manner (Vahrenkamp *et al.*, 1997 ▸), were isolated starting from **L^2^** and **L^3^**, respectively, under the same experimental conditions (Lippolis *et al.*, 1999 ▸). [Ag_2_(**L^1^**)_2_(μ-CN)]BF_4_·MeCN was the first discrete binuclear com­plex, and is still the only one reported in the literature, featuring a pure two-electron (σ) μ_2_-κ*C*:κ*C* bridging cyanide, to be structurally characterized. This result was initially attributed to the different length of the pendant arms in the macrocyclic ligands employed; the presence of shorter and less sterically demanding arms in **L^1^** as com­pared to **L^2^** would allow a closer approach of two [Ag(**L^1^**)]^+^ units in the binuclear com­plex featuring a side-on bridging cyanide. Herein we report a further development of this chemistry from a crystallographic point of view, with the aim of better understanding the role played by the length of the aliphatic chain in nitrile-functionalized derivatives of the small-ring macrocycles [9]aneN_2_S (1-thia-4,7-di­aza­cyclo­nona­ne) and [9]aneNS_2_ (1,4-di­thia-7-aza­cyclo­nona­ne) in determining the coordination chemistry towards silver(I) both in the absence or in the presence of exogenous bridging ligands. 
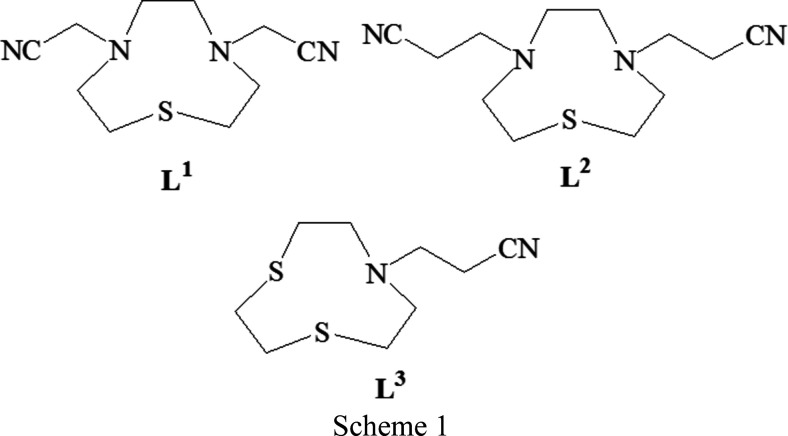


The com­pounds studied are [Ag(**L^1^**)_2_]BF_4_, [Ag(**L^2^**)(SCN)] and [Ag_2_(**L^3^**)_2_(μ-SCN)]BF_4_ (Scheme 2).

## Experimental

### Material and methods

All starting materials were obtained from Aldrich and were used without further purification. Microanalyses were per­formed by the University of Nottingham School of Chemistry Microanalytical Service. IR spectra were recorded as KBr discs using a PerkinElmer 598 spectrometer over the range 200–4000 cm^−1^. Fast atom bombardment (FAB) mass spectra were recorded at the EPSRC Centre for Mass Spectroscopy at the University of Swansea, UK.

### Synthesis and crystallization

4,7-Bis(cyano­meth­yl)-1-thia-4,7-di­aza­cyclo­nonane (**L^1^**), 4,7-bis­(2-cyano­eth­yl)-1-thia-4,7-di­aza­cyclo­nonane (**L^2^**) and 7-(2-cyano­eth­yl)-1,4-di­thia-7-aza­cyclo­nonane (**L^3^**) were prepared according to adaptations of procedures reported in the literature (Fortier & McAuley, 1989 ▸; Chak *et al.*, 1994 ▸). The experimental conditions considered for the reaction of **L^2^** and **L^3^** with silver(I) in the presence of thio­cyanate, namely, an **L**/Ag^+^/SCN^−^ molar ratio of 1:1:0.5, were the same as those used for the reactions in the presence of cyanate. In both cases, the aim was to favour the bridging coordination mode of the anionic ligand. 
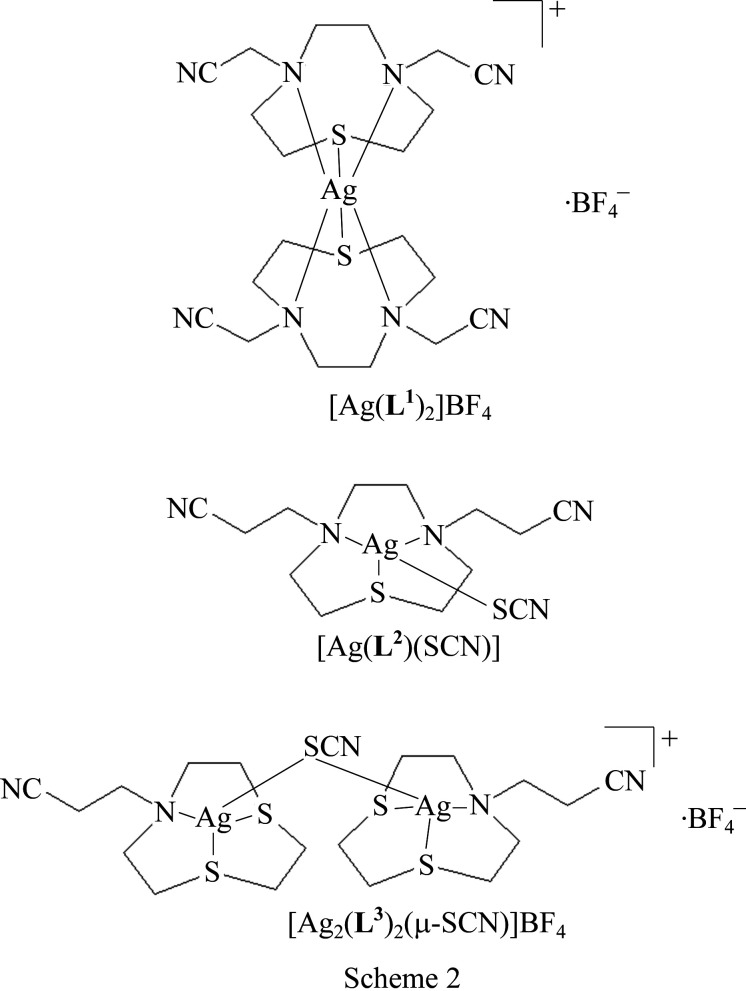


#### Synthesis of [Ag(**L^1^**)_2_]BF_4_

A mixture of 4,7-bis­(cyano­meth­yl)-1-thia-4,7-di­aza­cyclo­nonane (**L^1^**) (20 mg, 0.089 mmol) and AgBF_4_ (17.33 mg, 0.089 mmol) in MeCN (5 ml) was stirred in the dark at room temperature for 12 h. The solvent was partially removed under reduced pressure and Et_2_O vapour was allowed to diffuse into the remaining solution. Colourless block-shaped crystals of the desired com­plex were obtained (yield 15.2 mg, 53%; m.p. 160 °C, with decom­posi­tion). Analysis calculated (%) for [Ag(**L^1^**)_2_]BF_4_, C_20_H_32_AgBF_4_N_8_S_2_: C 37.34, H 5.01, N 17.42; found: C 37.28, H 4.99, N 17.20. FAB mass spectrum (3-NOBA) *m*/*z*: 555 and 331 for [^107^Ag(**L^1^**)_2_]^+^ and [^107^Ag(**L^1^**)]^+^, respectively. IR spectrum (KBr disc) ν (cm^−1^): 2928 (*m*), 2833 (*m*), 2243 (*s*) (ν_CN_ stretch in **L^1^**), 1452 (*s*), 1335 (*s*), 1223 (*w*), 1109 (*m*), 998 (*s*), 920 (*w*), 878 (*m*).

#### Synthesis of [Ag(**L^2^**)(SCN)]

A mixture of 4,7-bis(2-cyano­eth­yl)-1-thia-4,7-di­aza­cyclo­nonane (**L^2^**) (21.7 mg, 0.086 mmol) and AgBF_4_ (16.74 mg, 0.086 mmol) in MeCN (3 ml) was stirred in the dark at room temperature for 30 min. A solution of ^*n*^Bu_4_NSCN (12.92 mg, 0.043 mmol) in MeCN (2.5 ml) was then added and the resulting mixture was stirred for a further 30 min in the dark at room temperature. After partial removal of the solvent under reduced pressure and filtration through a pad of celite, colourless crystals were formed upon diffusion of Et_2_O vapour into the remaining solution (yield 10.5 mg; 58%; m.p. 135 °C, with decom­posi­tion). Analysis calculated (%) for [Ag(**L^2^**)(SCN)], C_13_H_20_AgN_5_S_2_: C 37.32, H 4.82, N 16.74; found: C 37.30, H 4.87, N 16.65. FAB mass spectrum (3-NOBA) *m*/*z*: 359 for [^107^Ag(**L^2^**)]^+^. IR spectrum (KBr disc) ν (cm^−1^): 2923 (*m*), 2824 (*m*), 2239 (*m*) (ν_CN_ stretch in **L^2^**), 2085 (*m*) (ν_CN_ stretch in SCN), 1472 (*m*), 1445 (*m*), 1415 (*m*), 1363 (*m*), 1306 (*w*), 1047 (*s*), 968 (*m*), 848 (*w*), 750 (*w*).

#### Synthesis of [Ag_2_(**L^3^**)_2_(μ-SCN)]BF_4_

A mixture of 7-(2-cyano­eth­yl)-1,4-di­thia-7-aza­cyclo­nonane (**L^3^**) (21.7 mg, 0.100 mmol) and AgBF_4_ (19.47 mg, 0.100 mmol) in MeCN (2.5 ml) was stirred in the dark at room temperature for 30 min. A solution of ^*n*^Bu_4_NSCN (15.027 mg, 0.050 mmol) in MeCN (2.5 ml) was then added and the resulting mixture was stirred for a further 30 min in the dark at room temperature. After partial removal of the solvent under reduced pressure and filtration through a pad of celite, colourless crystals were formed upon diffusion of Et_2_O vapour into the remaining solution (yield 18.3 mg; 46%; m.p. 140–142 °C). Analysis calculated (%) for [Ag_2_(**L^3^**)_2_(μ-SCN)]BF_4_, C_19_H_32_Ag_2_BF_4_N_5_S_5_: C 28.76, H 4.07, N 8.83; found: C 28.65, H 3.98, N 8.78. FAB mass spectrum (3-NOBA) *m*/*z*: 323 for [^107^Ag(**L^3^**)]^+^. IR spectrum (KBr disc) ν (cm^−1^): 2911 (*m*), 2826 (*m*), 2246 (*m*) (ν_CN_ stretch in **L^3^**), 2105 (*m*) (ν_CN_ stretch in SCN), 1462 (*m*), 1410 (*m*), 1361 (*m*), 1303 (*m*), 1037 (*s*), 958 (*w*), 940 (*w*), 899 (*w*), 830 (*w*), 810 (*w*), 710 (*w*).

### Refinement of X-ray data

Crystal data, data collection and structure refinement details are summarized in Table 1 ▸. Methyl­ene H atoms were refined as riding on their parent C atoms, with *U*_iso_(H) = 1.2*U*_eq_(C).

## Results and discussion

**Figure 1:**
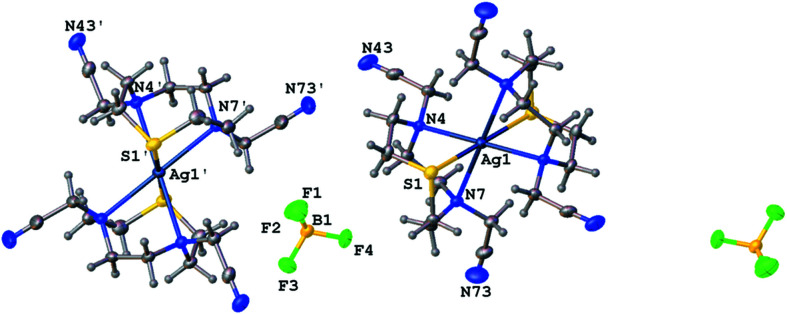
Two asymmetric units of com­plex [Ag(**L^1^**)_2_]BF_4_, showing the atom-numbering scheme. Displacement ellipsoids are drawn at the 50% probability level. The heteroatoms of the asymmetric unit are labelled.

Following the synthetic strategy adopted in previous studies to favour the formation of inorganic polymers, we reacted **L^1^** with AgBF_4_ in MeCN using a 1:1 metal-to-ligand molar ratio. Colourless tabular crystals formed after partial removal of the solvent and subsequent diffusion of Et_2_O vapour into the remaining solution. A single-crystal X-ray structure determination confirmed the product to be the discrete mononuclear Ag^I^ homoleptic com­plex [Ag(**L^1^**)_2_]BF_4_. Two ligands bind facially to the metal centre *via* the tridentate macrocyclic moiety, thus conferring a distorted octa­hedral coordination geometry of four N-donor and two S-donor atoms (Fig. 1 ▸), with no involvement of the nitrile groups from the pendant arms in metal coordination. The sandwich com­plex cations lie on crystallographic inversion centres, with the asymmetric unit consisting of two half-cations and one BF_4_^−^ anion (*Z* = 2). Each equatorial plane is defined by the N-donor atoms of two macrocyclic moieties [Ag1—N4 = 2.6173 (12), Ag1—N7 = 2.6822 (14), Ag1′—N4′ = 2.6363 (12) and Ag1′—N7′ = 2.6108 (13) Å], while the apical positions are occupied by the S-donor atoms [Ag1—S1 = 2.5273 (4) and Ag1′—S1′ = 2.5605 (4) Å] (Table 2 ▸). The Ag—N bond lengths are slightly longer than those reported for the sandwich com­plex [Ag(Me_3_[9]aneN_3_)_2_]PF_6_ [Ag—N = 2.543 (10) and 2.607 (7) Å; Me_3_[9]aneN_3_ = 1,4,7-trimethyl-1,4,7-tri­aza­cyclo­nonane] (Stock­heim *et al.*, 1991 ▸), while the Ag—S bond length is significantly shorter than those observed in the sandwich com­plex [Ag([9]aneS_3_)_2_](CF_3_SO_3_) [2.696 (2)–2.753 (1) Å; [9]aneS_3_ = 1,4,7-ththia­cyclo­nona­ne] (Blower *et al.*, 1989 ▸).

**Figure 2:**
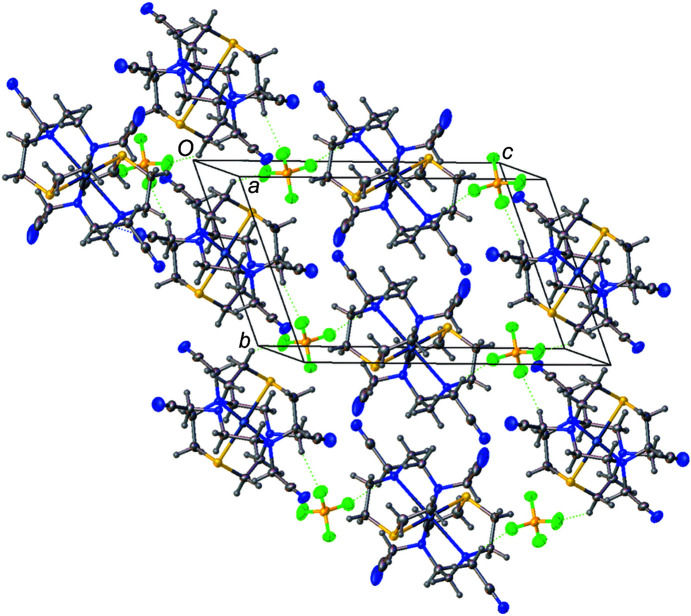
The extended structure of [Ag(**L^1^**)_2_]BF_4_, viewed approximately along the *a* axis. The structure features C—H⋯F and C—H⋯N inter­actions (shown as dotted lines), which link cations and anions into layers.

The extended structure of [Ag(**L^1^**)_2_]BF_4_ features C—H⋯N and C—H⋯F inter­actions characterized by H⋯*A* distances of 2.36–2.62 Å and *D*—H⋯*A* angles of 128–161° (see Table S1 in the supporting information). These inter­actions link cations and anions into chains (see Fig. 2 ▸; com­plementary views of the packing are available as Figs. S1 and S2 in the supporting information) and crosslink these chains to form layers.

The formation of the mononuclear sandwich com­plex [Ag(**L^1^**)_2_]BF_4_ upon reaction of **L^1^** with silver(I) appears to support the hypothesis that longer nitrile pendant arms favour the formation of polynuclear com­plexes *via* bridging different metal centres that occupy different ring cavities. Thus, reaction of **L^2^** with AgBF_4_ afforded the binuclear com­plex [Ag_2_(**L^2^**)_2_[(BF_4_)_2_, in which two inversion-related [Ag(**L^2^**)]^+^ units are held together by Ag—N bonds involving one nitrile-functionalized pendant arm from each ligand; the remaining two pendant arms are uncoordinated (Tei *et al.*, 2002 ▸). Also, the formation of a sinusoidal one-dimensional polymer is observed in {[Ag(**L^3^**)]BF_4_}_ 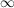 _, in which each Ag^I^ ion of the [Ag(**L^3^**)]^+^ repeating unit is bound by the [9]aneN_2_S macrocyclic moiety of the ligand and by the nitrile group of a symmetry-related [Ag(**L^3^**)]^+^ unit (Tei *et al.*, 2002 ▸).

The observed steric influence of nitrile-functionalized pendant arms on the formation of a polynuclear silver(I) com­plex cannot be the same in the presence of exogenous bridging ligands. The results obtained in the presence of CN^−^ {side-on coordination in the case of the binuclear com­plex cation [Ag_2_(**L^1^**)_2_(μ-CN)]^+^ and end-on coordination in the case of [Ag_2_(**L^2^**)_2_(μ-CN)]^+^ and [Ag_2_(**L^3^**)_2_(μ-CN)]^+^} seem to indicate the same trend observed in the absence of the *pseudo*-halogen (Lippolis *et al.*, 1999 ▸). In order to test this idea, we considered the reaction of **L^2^** and **L^3^** with NCS^−^ that, like CN^−^, can coordinate to metals in both terminal and bridging modes; moreover, as bridging ligands, NCS^−^ can also link metal centres in either an end-on or a side-on bonded fashion.

**Figure 3:**
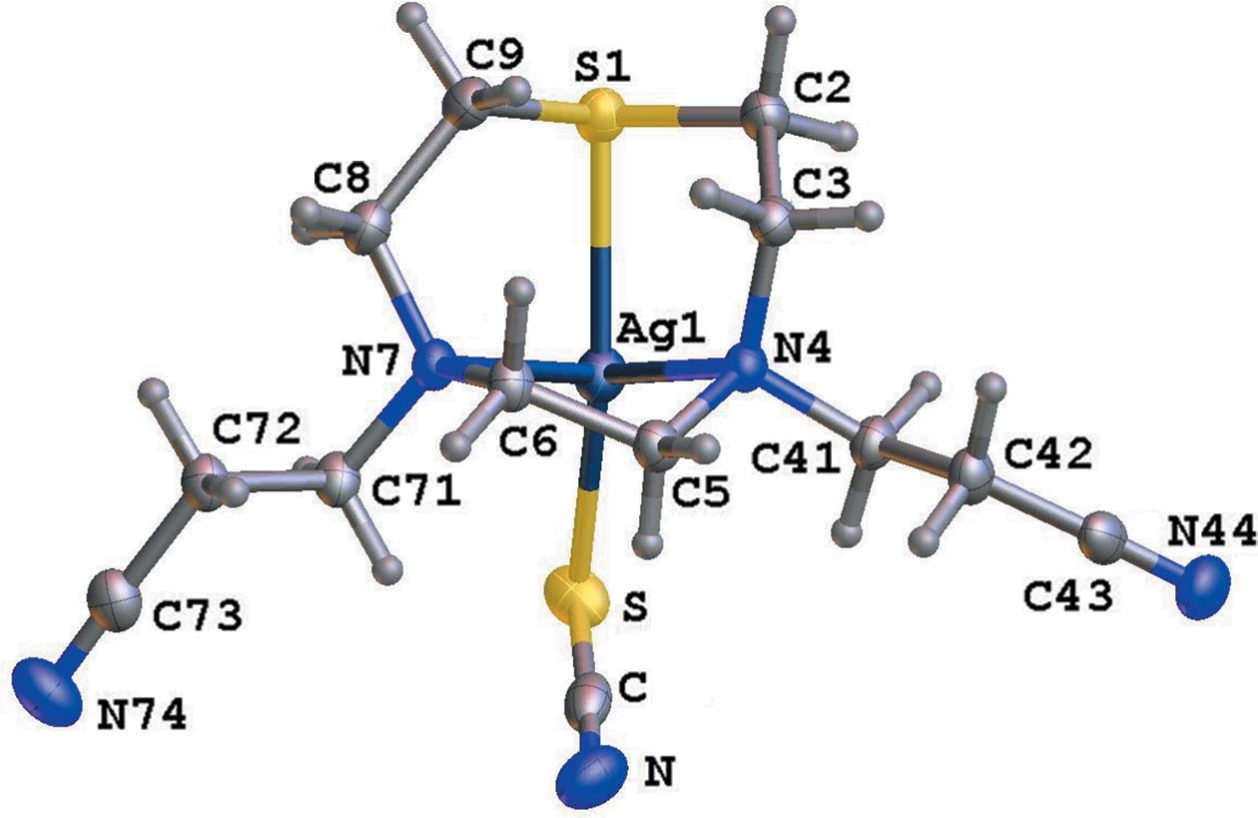
The asymmetric unit of com­plex [Ag(**L^2^**)(SCN)]BF_4_, showing the atom-numbering scheme. Displacement ellipsoids are drawn at the 50% probability level.

Reaction of **L^2^** with 1 equiv. of AgBF_4_ in MeCN in the presence of 0.5 equiv. of ^*n*^Bu_4_NSCN was carried out under the same experimental conditions used for the reaction performed in the presence of CN^−^. After partial removal of the solvent and filtration through a pad of celite, colourless crystals were formed upon diffusion of Et_2_O vapour into the remaining solution. Microanalytical data suggested the formulation [Ag(**L^2^**)(SCN)] for the product obtained and an X-ray diffraction analysis was undertaken to elucidate the coordination mode of the ligand NCS^−^. The com­pound consists of mononuclear units and shows the metal centre facially coordinated to the macrocyclic moiety of **L^2^** [Ag1—N4 = 2.5490 (14), Ag1—N7 = 2.5561 (15) and Ag1—S1 = 2.5074 (5) Å] and to a terminal SCN^−^ anion ligand *via* its S-donor atom [Ag1—S 2.4390 (5) Å] in a tetra­hedral geom­etry (Fig. 3 ▸).

**Figure 4:**
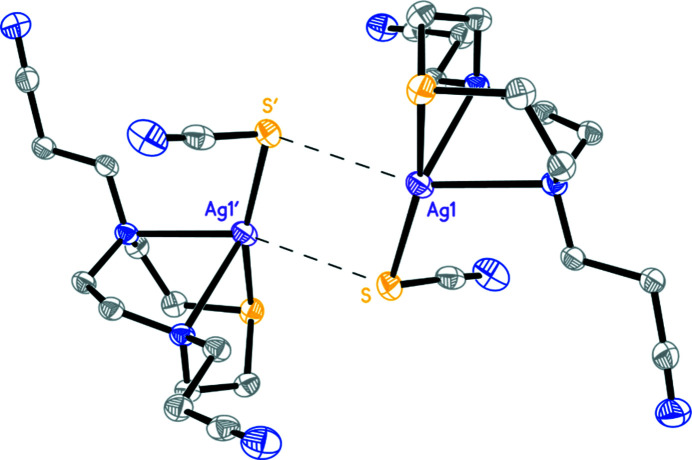
View of a pair of [Ag(**L^2^**)(SCN)] mol­ecules, showing inter­molecular Ag⋯S inter­actions. H atoms have been omitted for clarity. [Symmetry code: (′) −*x* + 1, −*y* + 1, −*z* + 1.]

The structure is very similar to that observed for [Ag([9]aneN_2_S)Cl] (Heinzel & Mattes; 1992 ▸), in which the Ag—N and Ag—S distances [Ag—N = 2.414 (5) and Ag—S = 2.629 (2) Å] are significantly shorter and longer, respectively, than those observed in [Ag(**L^2^**)(SCN)] and in [Ag(Me_3_[9]aneN_3_)(SCN)] (Stockheim *et al.*, 1991 ▸). The Ag—N distances in [Ag(Me_3_[9]aneN_3_)(SCN)] are com­parable with those in [Ag([9]aneN_2_S)Cl]. As observed in [Ag(Me_3_[9]aneN_3_)(SCN)], the structure of [Ag(**L^2^**)(SCN)] shows mol­ecular com­plex units packed pairwise, with the silver(I) ion and the S-donor from the thio­cyanate ligand of two different com­plex units inter­acting in a head-to-tail manner to form a planar four-membered rhombohedral ring with inter­molecular Ag⋯S inter­actions of 3.2421 (6) Å (Fig. 4 ▸).

**Figure 5:**
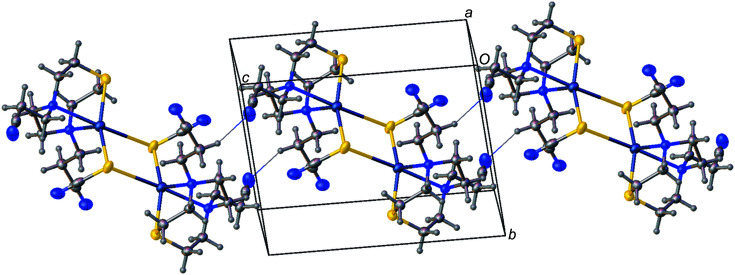
Partial view, approximatively along the *a* axis, of a chain of pairs of [Ag(**L^2^**)(SCN)] mol­ecules linked *via* C—H⋯N(nitrile) inter­actions.

Presumably these inter­actions, rather than steric factors, are responsible for the fact that the nitrile groups are not involved in metal coordination and the thio­cyanate ligand prefers to coordinate the metal centre in terminal rather than in bridging mode. Furthermore, pairs of [Ag(**L^2^**)(SCN)] com­plex units are linked into chains of mol­ecules by C42—H42*A*⋯N74^i^ inter­actions [N74^i^⋯H42*A* = 2.34 Å and C42—H42*A*⋯N74^i^ = 157°; symmetry code: (i) −*x* + 1, −*y* + 1, −*z*] (Fig. 5 ▸). These chains run parallel to the *c* axis. The C—H⋯N(nitrile) inter­actions are supported by C—H⋯N(thio­cyanate) inter­actions (not shown for clarity in Fig. 5 ▸) [C72—H72*A*⋯N^i^: N^i^⋯H72*A* = 2.51 Å and C72—H72*A*⋯N^i^ = 142°; see Table S2 in the supporting information for short contacts in the structure].

**Figure 6:**
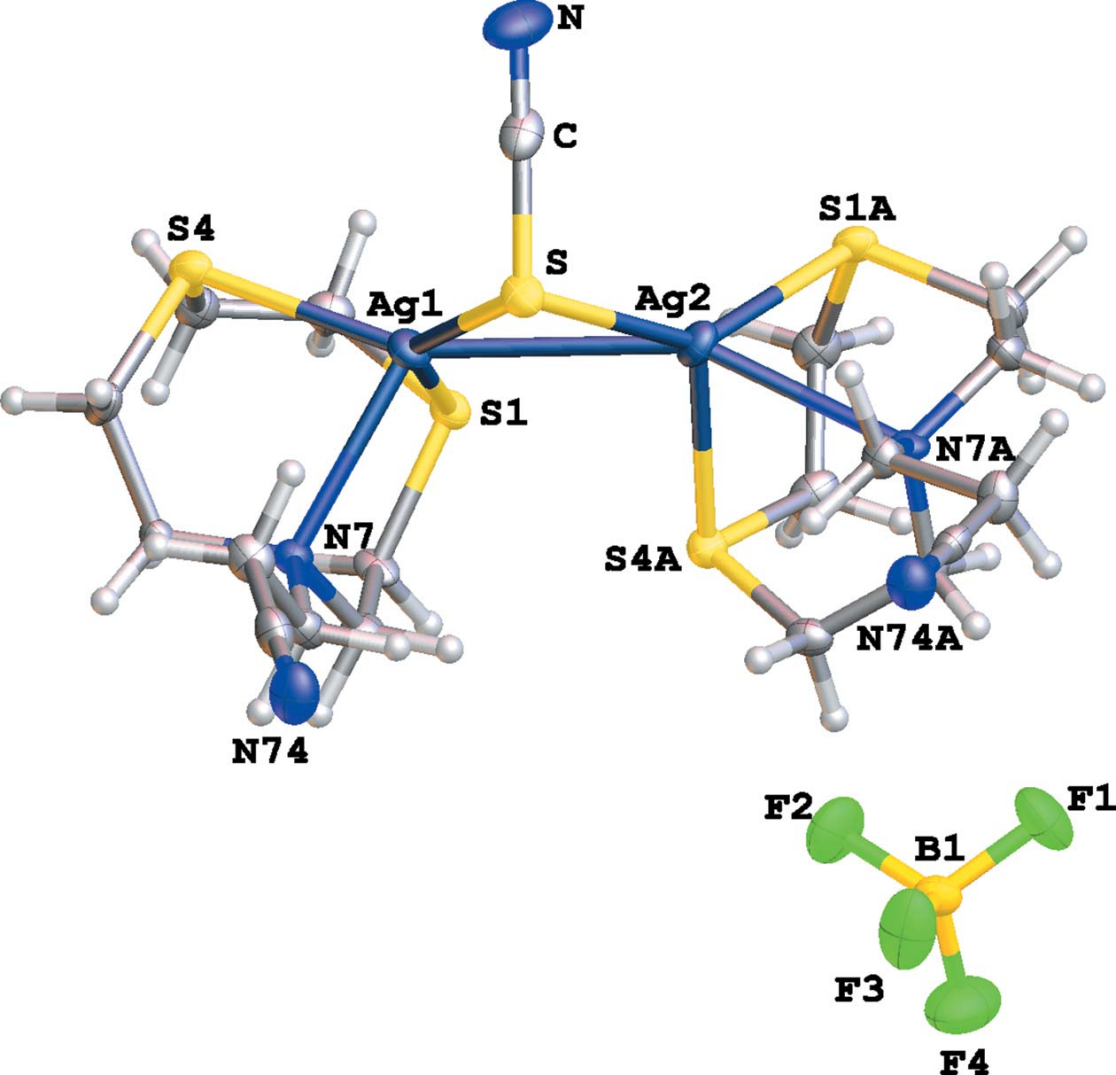
The asymmetric unit of com­plex [Ag_2_(**L^3^**)_2_(μ-SCN)]BF_4_, showing the atom-numbering scheme. Displacement ellipsoids are drawn at the 50% probability level.

Surprisingly, the reaction of **L^3^** with AgBF_4_ and ^*n*^Bu_4_NSCN under the same experimental conditions used for [Ag(**L^2^**)(SCN)] afforded the binuclear com­plex [Ag_2_(**L^3^**)_2_(μ-SCN)]BF_4_, which shows a μ_2_-κ*S*:κ*S* bridging NCS^−^ ligand acting as a σ two-electron donor between two metal centres of [Ag(**L^3^**)]^+^ com­plex cationic units [Ag1—S = 2.4943 (13) and Ag2—S = 2.4441 (13) Å] (Fig. 6 ▸).

The structure is very similar to that observed for the [Ag_2_(**L^1^**)_2_(μ-CN)]^+^ com­plex cation except that **L^1^** (which has two –CH_2_CN pendant arms) and CN^−^ are replaced by **L^3^** (which has only one –CH_2_CH_2_CN pendant arm) and NCS^−^. The Ag1—S—Ag2 angle of 76.91 (4)° is slightly smaller than the angle at the side-on bridging cyanide [Ag1—C—Ag2 = 79.5 (3)°] in [Ag_2_(**L^1^**)_2_(μ-CN)]^+^ (Lippolis *et al.*, 1999 ▸), with the Ag—Ag distance being significantly longer [3.0716 (6) Å] com­pared to the value of 2.7557 (10) Å in [Ag_2_(**L^1^**)_2_(μ-CN)]^+^; this could be a consequence of the longer Ag—S distances com­pared to Ag—C [2.153 (8) and 2.155 (8) Å, respectively]. The packing in [Ag_2_(**L^3^**)_2_(μ-SCN)]BF_4_ is a 3D network built up by an array of C—H⋯N, C—H⋯F and C—H⋯S inter­actions (see Fig. S3 and Table S3 in the supporting information).

The com­plex cation [Ag_2_(**L^3^**)_2_(μ-SCN)]^+^ represents the first discrete binuclear silver(I) com­plex featuring a two-electron (σ) μ_2_-κ*S*:κ*S* bridging thio­cyanate. A similar coordination mode of SCN^−^ in discrete binuclear com­plexes has only been observed in the com­plex anion [Hg_2_(SCN)_7_]^3−^ in [Co(NH_3_)_6_][Hg_2_(SCN)_7_] (Bala *et al.*, 2006 ▸).

## Conclusions

In this article, we have described the crystal structures of three new silver(I) com­plexes of nitrile-functionalized pendant-arm derivatives of the tridentate macrocyclic ligands [9]aneN_3_, [9]aneN_2_S and [9]aneNS_2_, including the presence of thio­cyanate (NCS^−^). The results obtained, as com­pared to those previously reported in the presence of cyanate (CN^−^), allow a better understanding of the role played by the number and length of the pendant arms in the coordination chemistry of this type of ligand towards silver(I). In general, longer more sterically-demanding nitrile-functionalized pendant arms in the macrocyclic derivatives (**L**) do not appear to prevent CN^−^ or NCS^−^ forming a side-on two-electron (σ) bridge rather than a linear four-electron (σ + π) one between two [Ag(**L**)]^+^ units, provided the appropriate pseudo-halide is used, *i.e.* steric factors appear not to be responsible for the fact that CN^−^ shows a linear μ_2_-κ*C*:κ*N* bridging mode in [Ag_2_(**L^3^**)_2_(μ-CN)]BF_4_, whereas NCS^−^ forms a side-on μ_2_-κ*S*:κ*S* bridge in the binuclear com­plex [Ag_2_(**L^3^**)_2_(μ-SCN)]BF_4_. In fact, steric factors cannot be considered solely responsible for this because an end-on μ_2_-κ*S*:κ*N* bridging mode for NCS^−^ would have allowed the two [Ag(**L^3^**)]^+^ units to dispose themselves further apart than in the case of [Ag_2_(**L^3^**)_2_(μ-CN)]^+^ where the shorter CN^−^ acts as a linear μ_2_-κ*C*:κ*N* bridging donor. On the other hand, with **L^2^** presenting two longer pendant arms as in **L^3^**, a linear μ_2_-κ*C*:κ*N* bridging mode is observed in [Ag_2_(**L^2^**)_2_(μ-CN)]BF_4_ for the cyanide ligand, while a terminal coordination mode is observed for NCS^−^ in the mononuclear tetra­hedral com­plex [Ag(**L^2^**)(SCN)]. A side-on μ_2_-κ*C*:κ*N* bridging mode is observed in [Ag_2_(**L^1^**)_2_(μ-CN)]BF_4_, where the macrocyclic ligand **L^1^** incorporates shorter pendant arms com­pared to **L^2^** and **L^3^**. This result suggests that some steric effects might also come into play, in combination with electronic requirements, in the coordination chemistry of nitrile-functionalized pendant arm derivative of small tridentate macrocycles with silver(I) in the presence of anionic ligands CN^−^ and NCS^−^.

## Supplementary Material

Crystal structure: contains datablock(s) AgL12BF4, AgL2SCN, Ag2L32mu-SCNBF4, global. DOI: 10.1107/S205322962200105X/ky3213sup1.cif

Structure factors: contains datablock(s) AgL12BF4. DOI: 10.1107/S205322962200105X/ky3213AgL12BF4sup2.hkl

Structure factors: contains datablock(s) AgL2SCN. DOI: 10.1107/S205322962200105X/ky3213AgL2SCNsup3.hkl

Structure factors: contains datablock(s) Ag2L32mu-SCNBF4. DOI: 10.1107/S205322962200105X/ky3213Ag2L32mu-SCNBF4sup4.hkl

Supporting information file. DOI: 10.1107/S205322962200105X/ky3213sup5.pdf

CCDC references: 2145502, 2145501, 2145500

## Figures and Tables

**Table 1:** Experimental details Experiments were carried out at 150 K with Mo *K*α radiation. H-atom parameters were constrained.

	[Ag(**L^1^**)_2_]BF_4_	[Ag(**L^2^**)(SCN)]	[Ag_2_(**L^3^**)_2_(μ-SCN)]BF_4_
Crystal data			
Chemical formula	[Ag(C_10_H_16_N_4_S)_2_]BF_4_	[Ag(C_12_H_20_N_4_S)(NCS)]	[Ag_2_(C_9_H_16_N_2_S_2_)_2_(SCN)]BF_4_
*M*_r_	643.33	418.33	793.34
Crystal system, space group	Triclinic, *P* 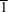	Triclinic, *P* 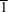	Monoclinic, *C*2/*c*
*a*, *b*, *c* (Å)	10.1813 (6), 10.2237 (6), 15.0154 (9)	8.4426 (6), 8.5739 (6), 11.8497 (8)	28.537 (3), 8.4362 (11), 27.216 (3)
α, β, γ (°)	73.446 (2), 82.398 (2), 61.781 (2)	96.585 (1), 99.023 (1), 95.313 (1)	90, 119.940 (9), 90
*V* (Å^3^)	1320.11 (14)	836.19 (1)	5677.6 (12)
*Z*	2	2	8
μ (mm^−1^)	0.98	1.46	1.79
Crystal size (mm)	0.3 × 0.2 × 0.14	0.36 × 0.30 × 0.07	0.27 × 0.15 × 0.12

Data collection			
Diffractometer	Bruker SMART CCD area-detector	Bruker SMART1000 CCD area-detector	Stoe STADI4 4-circle
Absorption correction	Multi-scan (*SADABS*; Bruker, 1996 ▸)	Integration (*SHELXTL*; Sheldrick, 2008 ▸)	Integration (*SHELXTL*; Sheldrick, 2008 ▸)
*T*_min_, *T*_max_	0.729, 0.828	0.606, 0.819	0.713, 0.822
No. of measured, independent and observed [*I* > 2σ(*I*)] reflections	13703, 6151, 5420	5159, 3668, 3401	5547, 4972, 4138
*R*_int_	0.035	0.022	0.025
(sin θ/λ)_max_ (Å^−1^)	0.679	0.675	0.594

Refinement			
*R*[*F* ^2^ > 2σ(*F* ^2^)], *wR*(*F* ^2^), *S*	0.023, 0.062, 1.07	0.021, 0.055, 1.06	0.036, 0.075, 1.15
No. of reflections	6151	3668	4972
No. of parameters	328	190	326
Δρ_max_, Δρ_min_ (e Å^−3^)	0.46, −0.34	0.34, −0.41	0.56, −0.52

Computer programs: *SMART* (Bruker, 1998 ▸), *STADI-4* (Stoe & Cie, 1996 ▸), *SAINT* (Bruker, 1999 ▸), *X-RED* (Stoe & Cie, 1996 ▸), *SHELXS97* (Sheldrick, 2008 ▸), *SHELXL2018* (Sheldrick, 2015 ▸), *OLEX2* (Dolomanov *et al.*, 2009 ▸) and *Mercury* (Macrae *et al.*, 2020 ▸).

**Table 2:** Selected geometric parameters (Å, °)

[Ag(**L^1^**)_2_]BF_4_			
Ag1—S1	2.5273 (4)	Ag1′—S1′	2.5605 (4)
Ag1—N4	2.6173 (12)	Ag1′—N4′	2.6363 (12)
Ag1—N7	2.6822 (14)	Ag1′—N7′	2.6108 (13)
			
S1—Ag1—N4	77.67 (3)	S1′—Ag1′—N4′	76.92 (3)
S1—Ag1—N7	76.40 (3)	S1′—Ag1′—N7′	76.70 (3)
N4—Ag1—N7	68.50 (4)	N7′—Ag1′—N4′	69.72 (4)
			
[Ag(**L^2^**)(SCN)]			
Ag1—S1	2.5074 (5)	Ag1—N7	2.5561 (15)
Ag1—S	2.4390 (5)	C—N	1.152 (3)
Ag1—N4	2.5490 (14)	C—S	1.670 (2)
			
S1—Ag1—N4	79.60 (4)	S—Ag1—N7	111.02 (4)
S1—Ag1—N7	79.77 (4)	N4—Ag1—N7	71.15 (5)
S—Ag1—S1	160.21 (2)	N—C—S	177.7 (2)
S—Ag1—N4	119.16 (4)		
			
[Ag_2_(**L^3^**)_2_(μ-SCN)]BF_4_			
Ag1—Ag2	3.0716 (6)	Ag2—S1*A*	2.5329 (13)
Ag1—S4	2.6065 (13)	Ag2—S4*A*	2.6046 (13)
Ag1—S1	2.5966 (13)	Ag2—S	2.4441 (13)
Ag1—S	2.4943 (13)	Ag2—N7*A*	2.557 (4)
Ag1—N7	2.492 (4)		
			
S1—Ag1—S4	83.55 (4)	S1*A*—Ag2—S4*A*	86.88 (4)
S—Ag1—S4	129.95 (4)	S1*A*—Ag2—N7*A*	78.06 (9)
S—Ag1—S1	142.23 (4)	S4*A*—Ag2—Ag1	85.08 (3)
N7—Ag1—Ag2	120.33 (9)	S—Ag2—S1*A*	143.47 (4)
N7—Ag1—S4	80.45 (9)	S—Ag2—S4*A*	124.09 (4)
N7—Ag1—S1	79.96 (9)	S—Ag2—N7*A*	123.39 (9)
N7—Ag1—S	117.91 (9)	N7*A*—Ag2—S4*A*	77.95 (9)
Ag1—S—Ag2	76.91 (4)		
