# Supplementary material for: Coordination chemistry of nitrile-functionalized mixed thia-aza macrocycles [9]aneN2S and [9]aneNS2 towards silver(I)
Source: Acta Crystallogr C Struct Chem. 2022 Feb 14;78(Pt 3):169–75. doi: 10.1107/S205322962200105X (PMC8896526; doi:10.1107/S205322962200105X)
Supplement: Supplementary file 5 [file c-78-00169-sup5.pdf]

STRUCTURAL CHEMISTRY

Volume 78 (2022)

Supporting information for article:

Coordination chemistry of nitrile-functionalized mixed thia-aza macrocycles [9]aneN_2S and [9]aneNS_2 towards silver(I)

Alexander J. Blake, Vito Lippolis and Martin Schroder

Supplementary Information

Contents

Table S1. Short contacts in structure [Ag(L^{1})$_2$]BF$_4$
Table S2. Short contacts in structure [Ag(L^{2})SCN]BF$_4$
Table S3. Short contacts in structure [Ag$_2$(L^{3})$_2$(μ-SCN)]BF$_4$
Figure S1. The extended structure of [Ag(L^{1})$_2$]BF$_4$ viewed approximately along the *b* axis.
Figure S2. The extended structure of [Ag(L^{1})$_2$]BF$_4$ viewed approximately along the *c* axis.
Figure S3. The extended structure of [Ag$_2$(**L**^{3})$_2$(μ-SCN)]BF$_4$ viewed approximately along the *a*, *b* and *c* axes.

Table S1. Short contacts in structure [Ag(**L**^{1})$_2$]BF$_4$

D = donor atom, H = hydrogen atom, A = acceptor atom

**D-H···A**	**D-H**	**H···A**	**D···A**	**D-H···A**	**Symm(A)**
C2'-H2'B···N43	0.99	2.56	3.385(2)	141	(1-x,1-y,1-z)
C2-H2B···N73'	0.99	2.62	3.321(3)	128	
C5-H5A···F1	0.99	2.45	3.170(2)	129	(1-x,1-y,2-z)
C6'-H6'B···N73	0.99	2.47	3.398(3)	156	(x,y,-1+z)
C8-H8B···F4	0.99	2.37	3.197(2)	140	
C41'-H41A···F2	0.99	2.36	3.274(2)	154	(-x,2-y,1-z)
C41-H41D···F3	0.99	2.43	3.272(2)	143	(1+x,-1+y,z)
C71'-H71B···F2	0.99	2.43	3.271(2)	143	
C71-H71C···F3	0.99	2.38	3.329(2)	161	(1-x,2-y,2-z)

Table S2. Short contacts in structure [Ag(**L**^{2})SCN]BF$_4$

D = donor atom, H = hydrogen atom, A = acceptor atom

**D-H···A**	**D-H**	**H···A**	**D···A**	**D-H···A**	**Symm(A)**
C42-H42B···N74	0.99	2.34	3.276(3)	157	(1-x,1-y,-z)
C72-H72A···N	0.99	2.51	3.345(3)	142	(1-x,1-y,-z)

Table S3. Short contacts in structure [Ag$_2$(**L**^{3})$_2$(μ-SCN)]BF$_4$

D = donor atom, H = hydrogen atom, A = acceptor atom

**D-H···A**	**D-H**	**H...A**	**D...A**	**D-H...A**	**Symm(A)**
C8A-H8AB···N	0.99	2.46	3.371(8)	152	(-x,y,1/2-z)
C5-H5A···F3	0.99	2.52	3.413(6)	151	(1/2-x,5/2-y,1-z)
C6-H6A···N74A	0.99	2.56	3.542(7)	170	(1/2-x,5/2-y,1-z)
C5A-H5AB···N74A	0.99	2.52	3.371(7)	144	(x,-1+y,z)
C9A-H9AA···F1	0.99	2.41	3.356(7)	159	(1/2-x,-1/2+y,1/2-z)
C9A-H9AB···S4	0.99	2.79	3.766(5)	171	(-x,y,1/2-z)
C2A-H2AB···N	0.99	2.51	3.361(7)	144	(-x,-1+y,1/2-z)
C71-H71A···N74	0.99	2.58	3.454(7)	147	(1/2-x,5/2-y,1-z)
C71A-H71C···N74	0.99	2.62	3.573(7)	162	(1/2-x,5/2-y,1-z)

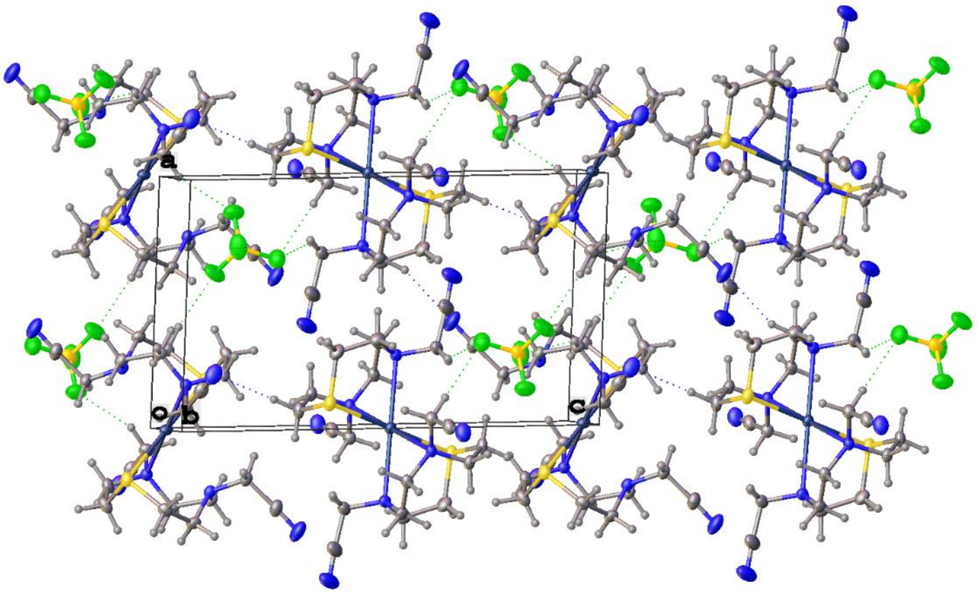

Figure S1. The extended structure of $[Ag(\mathbf{L}^1)_2]BF_4$ viewed approximately along the *b* axis.

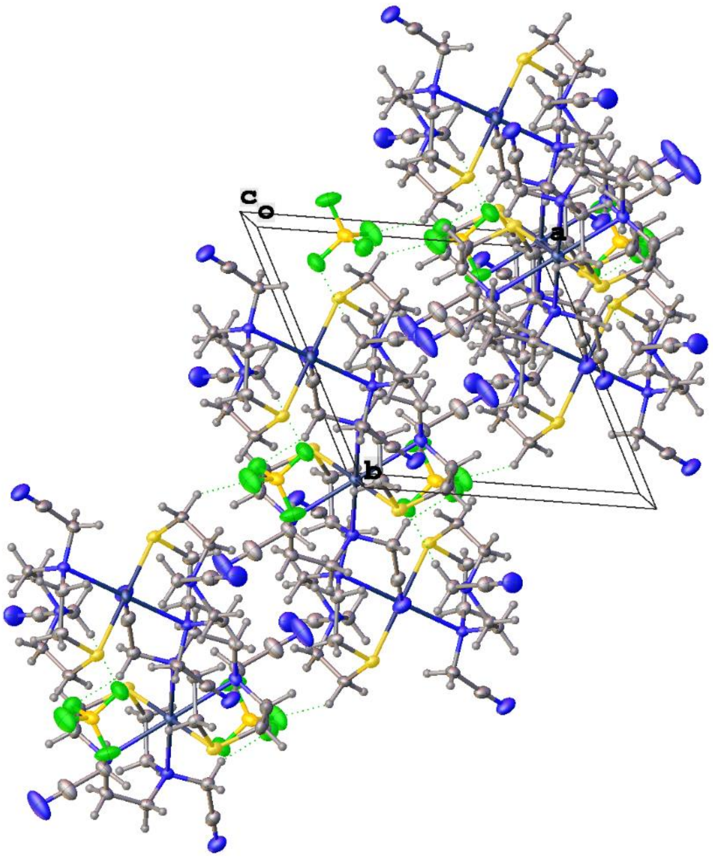

Figure S2. The extended structure of [Ag(**L**[1])$_2$]BF_4 viewed approximately along the *c* axis.

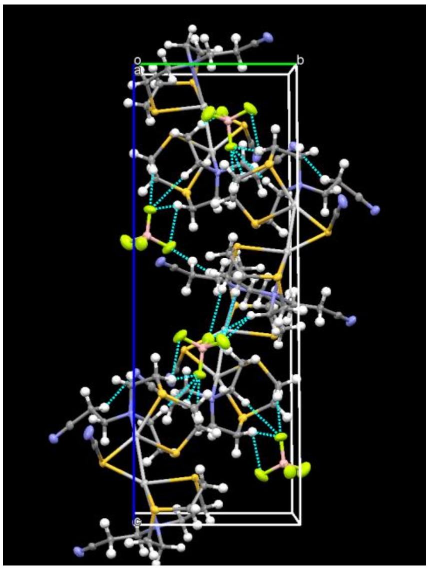

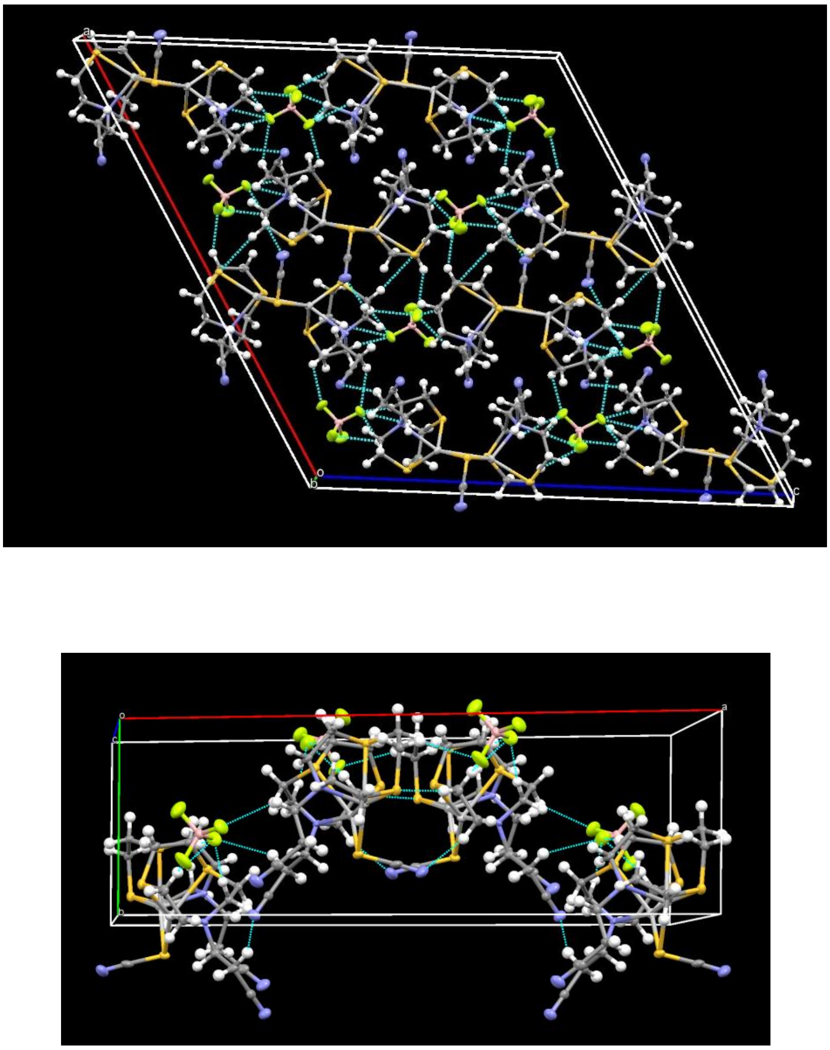

Figure S3. The extended structure of $[Ag_2(\mathbf{L}^3)_2(\mu\text{-SCN})]BF_4$ viewed approximately along the *a*, *b* and *c* axes. The depth of the views along *a* and *c* have been restricted because of the length of these axes.
